# Dishevelled-3 phosphorylation is governed by HIPK2/PP1Cα/ITCH axis and the non-phosphorylated form promotes cancer stemness via LGR5 in hepatocellular carcinoma

**DOI:** 10.18632/oncotarget.17049

**Published:** 2017-04-11

**Authors:** Yu-Man Tsui, Karen Man-Fong Sze, Edmund Kwok-Kwan Tung, Daniel Wai-Hung Ho, Terence Kin-Wah Lee, Irene Oi-Lin Ng

**Affiliations:** ^1^ State Key Laboratory for Liver Research, The University of Hong Kong, Hong Kong; ^2^ Department of Pathology, The University of Hong Kong, Hong Kong; ^3^ Present address: Department of Applied Biology and Chemical Technology, The Hong Kong Polytechnic University, Hong Kong

**Keywords:** Wnt/β-catenin, tumorigenicity, sphere formation, post-translational modification

## Abstract

Dishevelled-3 (Dvl3) is regarded as a binding hub with many different interacting partners. However, its regulation and mechanism on cancer stemness remain to be explored. In this study, we showed that Dvl3 was significantly overexpressed in human hepatocellular carcinomas (HCCs) and promoted cancer stemness both *in vitro* and *in vivo*. We found that the non-phosphorylated (NP)-Dvl3 was more stable than the phosphorylated form, more active in activating β-catenin transcriptional activity, and more potent in enhancing self-renewal ability in HCC cells. Mechanistically, we confirmed that the homeodomain-interacting protein kinase-2 (HIPK2) and E3 ubiquitin ligase ITCH were able to physically bind to Dvl3 protein. Knockdown of HIPK2 and the protein phosphatase regulatory unit C-alpha (PP1Cα) resulted in sustained Dvl3 phosphorylation and hence decrease in the NP form of Dvl3. On the other hand, knockdown of E3 ubiquitin ligase ITCH reduced the phosphorylation-induced degradation and stabilized the phosphorylated Dvl3 protein. Furthermore, the NP-Dvl3 enhanced the LGR5 promoter activity to upregulate LGR5 expression, which was associated with increased cancer stemness in HCC. Our findings established that HIPK2/PP1Cα/ITCH axis sustains the de-phosphorylation of Dvl3. This post-translational modification of Dvl3 in turn maintains LGR5 expression and enhances the cancer stemness properties in HCC.

## INTRODUCTION

Hepatocellular carcinoma (HCC) is the third leading cause of cancer deaths worldwide [1]. One of the frequently deregulated pathways in HCC is Wnt signaling. From our and others' previous results, somatic mutations of β-catenin are much less frequent (12-40%) [2] than β-catenin overexpression in human HCC (50-68%) [2, 3], suggesting that factors other than β-catenin mutation contribute to β-catenin activation.

Dishevelled (Dvl) protein is an important mediator of the Wnt/β-catenin signaling pathway. It is a family of three members, namely Dvl1, 2, and 3, and sharing three conserved domains, DIX, PDZ and DEP domains [4]. Dvl3 has been regarded as a binding hub with many different interacting partners [4]. Previously, we have demonstrated that Prickle-1 enhanced the ubiquitination and degradation of Dvl3 in HCC [5]. Recent studies suggested that the non-phosphorylated (NP) form of another Dvl family member, Dvl2, is more active in HEK293 cells [6] and the de-phosphorylation of Dvl protein promotes its stabilization in HeLa cells and zebra fish embryos [7]. In addition, it has been identified in HeLa cells and zebrafish embryos that the homeodomain-interacting protein kinase 2 (HIPK2) and protein phosphatase regulatory unit C-alpha (PP1Cα) are regulators of Dvl protein phosphorylation [7]: With HIPK2 bound to Dvl3, the phosphatase PP1Cα promotes the de-phosphorylation of phosphorylated Dvl3 and maintains Dvl3 protein in the non-phosphorylated form. This spares the Dvl3 protein from the phosphorylation-induced degradation by itchy E3 ubiquitin ligase (ITCH) [8].

LGR5 has been identified as a marker of crypt-locating intestinal stem cells [9, 10] and gastric stem cells [11]. It is a β-catenin target gene; as an intestine stem cell marker in the crypt of intestine [12, 13], it maintains tumor initiating cells in colorectal cancer and gastric cancer [12, 14, 15]. It can fuel the activation of Wnt/β-catenin signaling in the presence of R-spondin and Wnt ligands [16–18]. In HCC, damage-induced LGR5-positive cells can regenerate hepatocytes and bile ducts *in vivo* [19]. A previous report from Japan has demonstrated overexpression of LGR5 in HCC [17] and pro-survival property of LGR5 in HCC cells [20]. These suggest that LGR5 may enhance cancer stemness properties in HCC.

In this study, we found that, in human HCCs, Dvl3 was overexpressed at protein level as compared with the corresponding non-tumor livers. The NP form of Dvl3 had enhanced stability and activity in HCC cells in promoting sphere forming ability in HCC cells. Our data also suggest that HIPK2/PP1Cα/ITCH participates to mediate Dvl3 phosphorylation and degradation to upregulate LGR5 expression. This in turn promotes cancer stemness and HCC formation, as we demonstrated in *in vitro* sphere formation assays and *in vivo* tumorigenicity assays in immunodeficient NOD-SCID mice.

## RESULTS

### Dvl3 was overexpressed in human HCCs and its stability and activity were determined by its phosphorylation status in promoting sphere formation ability

**Figure 1:**
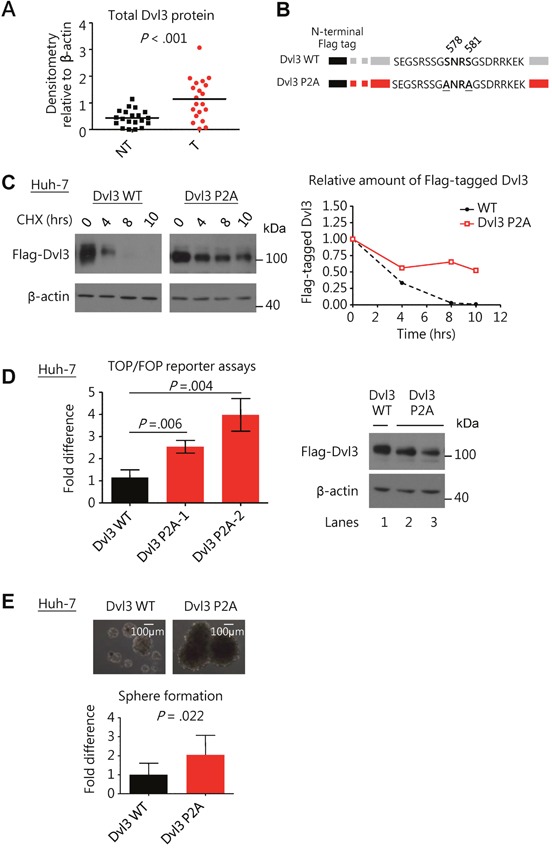
Dvl3 protein expression is enhanced in HCCs and the non-phosphorylated Dvl3 is the more stable and active form of Dvl3 in HCC cells to promote sphere formation **(A)** Scatter plot showing a summary of Dvl3 protein expression in 20 paired HCC (T) and corresponding non-tumorous tissues (NT) as analyzed by Western blot densitometry. **(B)** Schematic diagram showing mutation of serine residues 578 and 581 on N-terminal Flag-tagged Dvl3. **(C)** The NP-Dvl3 mutant showed sustained protein stability at different time points as compared to the WT upon treatment with cycloheximide (CHX) at 10 ug/ml in Huh-7. **(D)** The NP-Dvl3 mutant was more active than WT Dvl3 in Huh-7. To ensure normalization of the amount of Dvl3 protein *(right)* to allow comparison in TOP/FOP reporter assays, DNA constructs were transfected at the following amounts: 2.5 μg of Dvl3 WT, 0.875 μg and 1.0 μg of Dvl3 P2A for lanes 1, 2, and 3, respectively. **(E)** The NP-Dvl3 mutant promoted greater sphere forming ability than WT Dvl3 in Huh-7. All *in vitro* experiments were carried out in at least 3 independent trials and the values are represented as mean ± SD.

Using Western blot analysis, we observed significantly higher protein expression levels of Dvl3 in the HCC tumors than the corresponding non-tumorous livers in 20 randomly selected human HCC pairs (*P* < .001) (Figure 1A and Supplementary Figure 1A). Previous studies have demonstrated that phosphorylation of another Dvl family member, Dvl2, at its C-terminal tail suppressed the Wnt signaling activity in HEK293 cells [6]. As the concerned phosphorylation sites are conserved among Dvl protein family members [6], we investigated whether the phosphorylation of Dvl3 might also suppress its ability to activate Wnt/β-catenin signaling in HCC cells. We constructed the phosphorylation-defective mutants of Dvl3 by mutating the reported phosphorylation sites at serine residues 578 and 581 to alanine residues (P2A) (Figure 1B). Successful mutation of the concerned phosphorylation sites on the Flag-tagged Dvl3 protein resulted in the phospho-defective mutant. In the Western blot, the upper band was lost as compared to the wild-type counterpart (Supplementary Figure 1B). The upper band corresponds to the phosphorylated Dvl3 (P-Dvl3) protein as previously reported [6] and we were able to verify this using phosphatase treatment on the immuno-precipitated Dvl3 protein (Supplementary Figure 1C) and the administration of phosphatase inhibitor okadaic acid to the cell culture (Supplementary Figure 1D). Furthermore, we observed increased stability of the P2A phospho-defective mutant as compared to the wild-type, as shown upon treatment with cycloheximide (CHX) in Huh-7 (Figure 1C). This led to the accumulation of Dvl3 P2A as compared with the Dvl3 wild-type protein, and such accumulation was observed in multiple HCC cell lines, including Huh-7, PLC/PRF/5, BEL-7402 and SMMC-7721 (Supplementary Figure 1E). This phenomenon was irrespective of the type of protein tag used on the Dvl3 protein (Supplementary Figure 1F). In addition to its increased stability, the P2A mutant was more active than the wild-type in activating the β-catenin transcriptional activity, as shown in the TOP/FOP dual luciferase reporter assay (*P* < .010) (Figure 1D). Importantly, the Dvl3 P2A mutant also enhanced the sphere forming ability of Huh-7, PLC/PRF/5 and SMMC-7721 cells as compared to the wild-type (*P* = .022, *P* = .018, and *P* = .023, respectively) (Figure 1E and Supplementary Figure 1G). These findings demonstrated that the non-phosphorylated Dvl3 (NP-Dvl3) was more stable and active than the P-Dvl3 in HCC cells.

### Dvl3 protein dephosphorylation was governed by HIPK2-PP1Cα-ITCH axis in HCC

**Figure 2:**
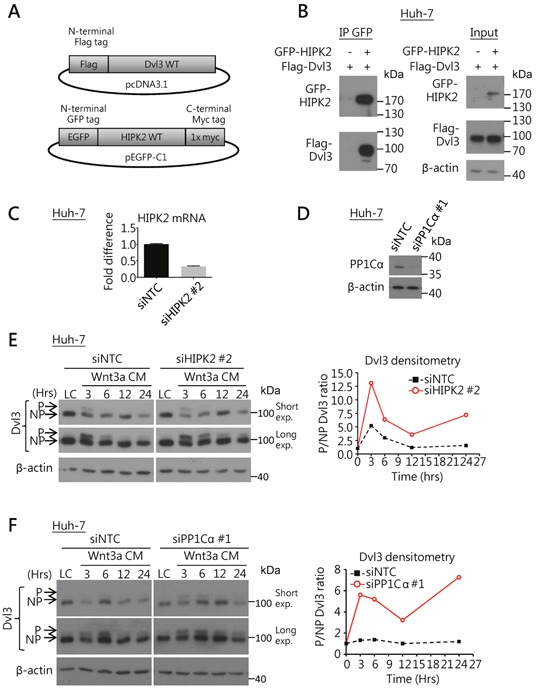
HIPK2/PP1Cα interacts with Dvl3 and is required for Dvl3 protein de-phosphorylation in HCC cells **(A)** Schematic diagrams for the DNA constructs used in the Co-IP experiment. **(B)** Co-IP experiment showed the binding of HIPK2 and Dvl3 proteins in Huh-7 cells. The result of one representative trial of the 3 independent experiments is shown. **(C)** Efficient knockdown of HIPK2 by siRNA in Huh-7 cells as compared to NTC. **(D)** Efficient knockdown of PP1Cα by siRNA in Huh-7 cells as compared to NTC. **(E)** Knockdown of HIPK2 sustained Wnt3a-induced phosphorylation of Dvl3 in Huh-7 cells. The cells were treated with Wnt3a conditioned medium (Wnt3a CM) for the indicated time periods upon transfection of the siRNA. Western blots of both short and long exposure (exp.) are shown *(left)*. Densitometry showing the ratio of the phosphorylated (P-) to the non-phosphorylated (NP-) Dvl3 (P/NP) plotted relative to the respective L-cell control medium (LC) upon the knockdown of HIPK2 *(right)*. **(F)** Knockdown of PP1Cα sustained the Wnt3a-induced phosphorylation of Dvl3 in Huh-7 cells. The cells were treated with Wnt3a-conditioned medium (Wnt3a CM) for the indicated periods of time upon transfection of the siRNA. Western blots of both short and long exposure (exp.) are shown *(left)*. Densitometry showing the ratio of the P- to the NP-Dvl3 (P/NP) plotted relative to the respective L-cell control medium (LC) upon the knockdown of PP1Cα *(right)*.

We explored the possible regulatory mechanism by HIPK2-PP1Cα-ITCH axis on Dvl3 in HCC cells. First, using co-immunoprecipitation (Co-IP) assay, we found that HIPK2 protein was able to physically bind to Dvl3 protein in Huh-7 cells (Figure 2A and 2B). We then used siRNA to knock down HIPK2 and PP1Cα, respectively, in Huh-7 cells (Figure 2C and 2D). Knockdown of HIPK2 sustained the Wnt3a-induced phosphorylation of Dvl3 protein at higher levels, in terms of the P-Dvl3 to NP-Dvl3 ratio, than the NTC (Figure 2E). Similarly, when the phosphatase PP1Cα was knocked down, the Wnt3a-induced Dvl3 phosphorylation, in terms of the ratio of P-Dvl3 to NP-Dvl3 protein levels, was increased and sustained for longer duration (Figure 2F). This indicates that HIPK2 and PP1Cα suppress Dvl3 phosphorylation and result in the increase of NP-Dvl3.

**Figure 3:**
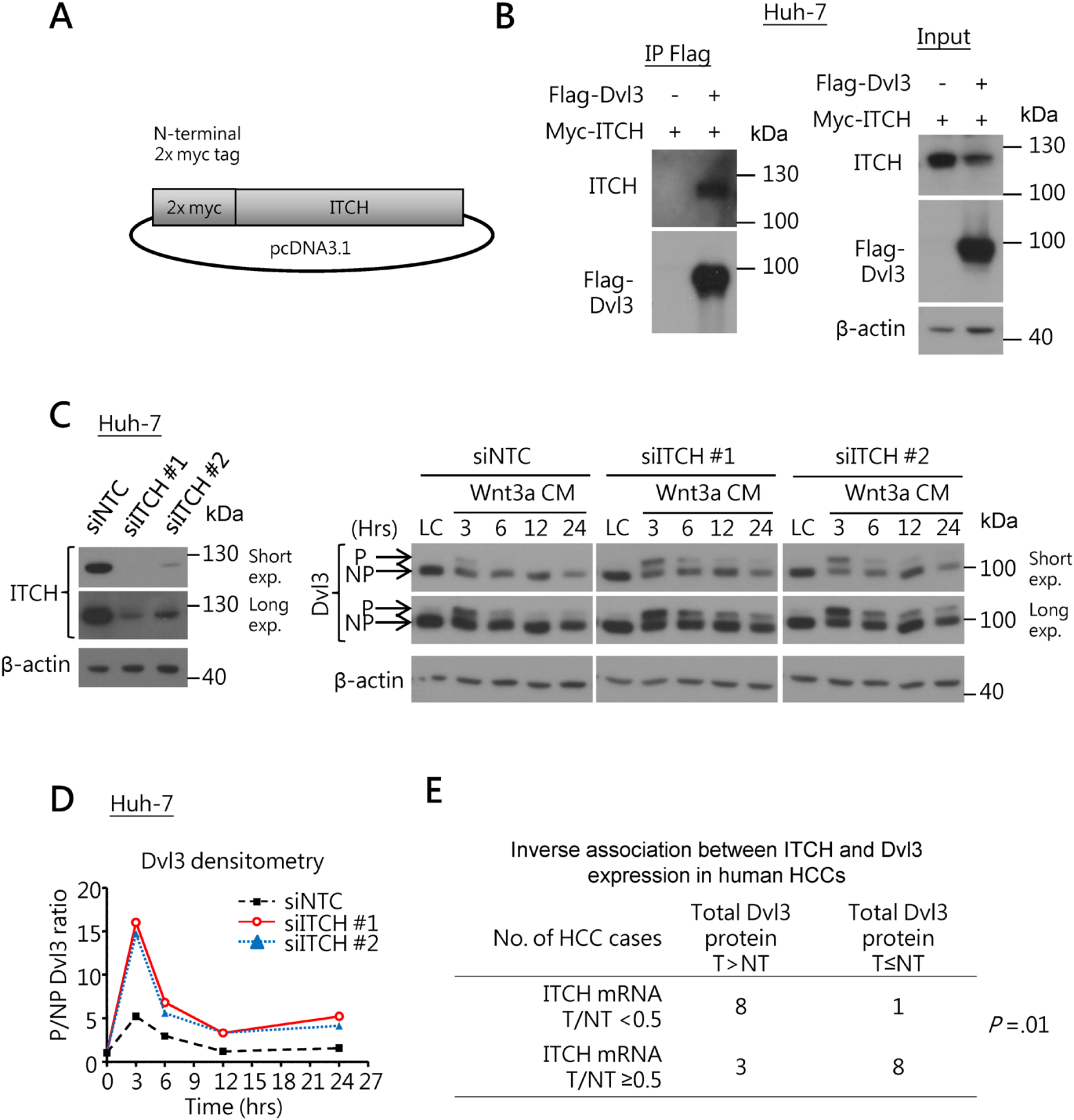
ITCH regulates Dvl3 protein expression in HCCs **(A)** Schematic diagram of the DNA construct of the N-terminally myc-tagged ITCH. **(B)** Co-IP experiment showed interaction between Dvl3 and ITCH proteins in Huh-7 cells. **(C)** Efficient knockdown of ITCH in Huh-7 using siRNA as compared to the NTC *(left)*. Knockdown of ITCH increased the level of Wnt3a-induced P-Dvl3 protein in Huh-7 cells *(right)*. The cells were treated with Wnt3a-conditioned medium (Wnt3a CM) for the indicated periods of time after transfection of the siRNA. Western blots of both short and long exposure (exp.) are shown. The result of one representative trial of the 3 independent experiments is shown. **(D)** Densitometry showing the P- to NP-Dvl3 ratio (P/NP) and plotted relative to the respective L-cell control medium (LC). **(E)** The ITCH mRNA level inversely correlated with the Dvl3 protein level in human HCCs.

Next, we examined whether ITCH also suppressed Dvl3 protein level in HCC, as suggested from the previous study on HEK293 cells [8]. Using Co-IP assay, we found that ITCH physically bound and interacted with Dvl3 protein in Huh-7 cells (Figure 3A and 3B). Knockdown of ITCH sustained the Wnt3a-induced P-Dvl3 in Huh-7 cells and this resulted in increase in the ratio of P-Dvl3 to NP-Dvl3 for longer duration than the NTC (Figure 3C and 3D). Of note, in human HCCs, the ITCH mRNA expression levels negatively correlated with the total Dvl3 protein (*P* = .01) (Figure 3E). The data support a role of ITCH in down-regulating the Dvl3 protein level in human HCCs.

### NP-Dvl3 and HIPK2-PP1Cα axis upregulated LGR5 expression in HCC cells

**Figure 4:**
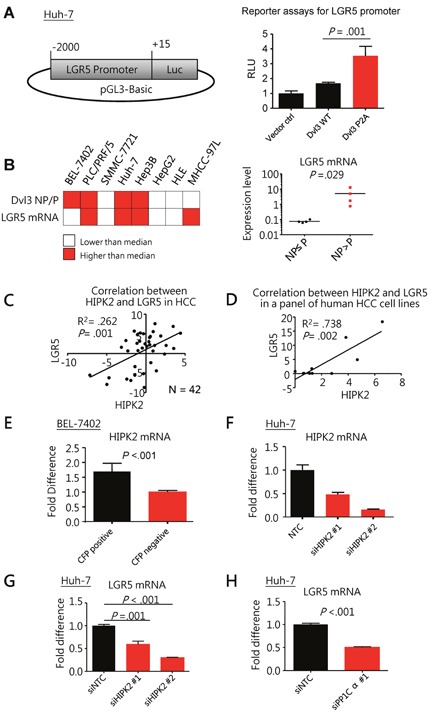
Non-phosphorylated Dvl3 as mediated by HIPK2/PP1Cα helps sustain LGR5 mRNA expression in HCC **(A)** Schematic diagram showing the cloning of LGR5 promoter to pGL3-Basic reporter plasmid used in the luciferase reporter assays *(left)*. The NP-Dvl3 mutant had greater LGR5 promoter activity than the WT Dvl3 *(right)* in Huh-7 cells. **(B)** Summary of Dvl3 protein phosphorylation status and LGR5 mRNA in HCC cell line panel *(left)*. The grey-shaded boxes represent values greater than the respective median among the cell lines while the white boxes indicate the opposite. Upon comparison of LGR5 expression levels between cell lines with NP>P Dvl3 and those with NP≤P Dvl3 *(right*), there was significant up-regulation of LGR5 expression in the former (P = 0.029, Mann Whitney test). Detailed values for individual cell lines can be found in Supplementary Figure 2D. **(C)** HIPK2 correlated with LGR5 at mRNA levels in our human HCC cohort. The fold difference of the mRNA expression between tumor and corresponding non-tumorous livers in terms of –ddCT for LGR5 and HIPK2 was plotted against each other to show their significant correlation in HCC by linear regression (N=42). Linear regression analysis was performed on HIPK2 and LGR5 mRNA levels. **(D)** HIPK2 correlated with LGR5 at mRNA levels in a panel of human HCC cell lines, including BEL-7402, PLC/PRF/5, SMMC-7721, Huh-7, Hep3B, HepG2, HLE and MHCC-97L. Linear regression analysis was performed on HIPK2 and LGR5 mRNA levels. Detailed values for individual cell lines can be found in Supplementary Figure 2D. **(E)** HIPK2 mRNA expression was significantly higher in LGR5-high cells than LGR5-low cells in BEL-7402. LGR5 promoter driven cyan-fluorescent protein (CFP) was used to indicate LGR5 expression and by fluorescence-activated cell sorting (FACS), the BEL-7402 cells with CFP signals and those without were sorted out. (**F** and **G**) Knockdown of HIPK2 by siRNA (F) significantly suppressed LGR5 expression in Huh-7 cells. **(H)** Knockdown of PP1Cα by siRNA significantly suppressed LGR5 expression in Huh-7 cells. All *in vitro* experiments were carried out in at least 3 independent trials and the values are represented as mean ± SD.

First, knockdown of Dvl3 in Huh-7 cells suppressed LGR5 expression whereas transient transfection of Dvl3 significantly increased it (Supplementary Figure 2A). Hence, we made use of LGR5 expression as a downstream readout for studying the activity of Dvl3 protein. With luciferase reporter assay, Dvl3 P2A mutant resulted in greater LGR5 promoter activity (2.1 folds) than the wild-type Dvl3 (*P* = .001) (Figure 4A). In addition, our panel of HCC cell lines showed a trend of positive correlation between NP-Dvl3 to P-Dvl3 ratio and LGR5 mRNA expression. Those cell lines with higher NP-Dvl3 to P-Dvl3 ratio (e.g. PLC/PRF/5, Huh-7 and Hep3B) had higher LGR5 mRNA expression (Figure 4B and Supplementary Figures 2B to 2D). On the contrary, those cell lines (e.g. SMMC-7721, HepG2 and HLE) with P-Dvl3 higher than NP-Dvl3 protein had almost undetectable LGR5 mRNA expression.

Furthermore, in human HCCs, the HIPK2 mRNA levels positively correlated with those of LGR5 (R^2^ = .262, *P* = .001) (Figure 4C). Besides our own data, we also found significant correlation between the mRNA levels of HIPK2 and LGR5 in HCC tumors in The Cancer Genome Atlas (TCGA) dataset (R^2^ = .427, *P* = .001) (Supplementary Figure 2E). Similar observation was also seen in our HCC cell line panel, the HIPK2 mRNA levels of which significantly correlated with those of LGR5 (R^2^ = .738, *P* = .002 by linear regression analysis) (Figure 4D). In order to investigate whether HIPK2 expression was specific to LGR5-high subpopulation within a cell line, we separated HCC cells with high LGR5 expression and those without by fluorescence-activated cell sorting (FACS). We found significantly enhanced HIPK2 expression in the HCC cells with high LGR5 expression (Figure 4E and Supplementary Figures 2F and 2G). These data indicated a significant association between HIPK2 and LGR5 expression and prompted us to further investigate whether HIPK2 expression could affect LGR5 expression.

We found that knockdown of HIPK2 (Figure 4F) significantly suppressed LGR5 expression in Huh-7 cells (*P* = .001) (Figure 4G). Correspondingly, knockdown of PP1Cα (Figure 2D) also significantly suppressed LGR5 expression (*P* < .001) (Figure 4H). This indicates that HIPK2 and PP1Cα regulate Dvl3 phosphorylation and also upregulate LGR5 expression in HCC cells.

### Dvl3, LGR5 and HIPK2 mediated stemness properties in HCC cells

**Figure 5:**
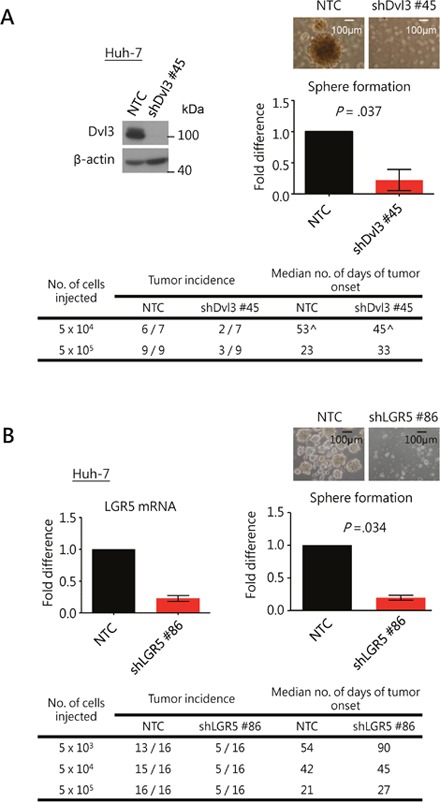
Knockdown of Dvl3 and LGR5 suppresses stemness properties in HCC cells **(A)** Efficient knockdown of Dvl3 was established in Huh-7 cell line using sh#45 as compared to the non-target control (NTC). This suppressed sphere formation *in vitro* significantly and inhibited tumorigenicity *in vivo*. The median number of days of tumor onset was calculated based on the successfully formed tumors. (^ The numbers of days after the injection for the onset of the six tumors in the NTC group were 28, 46, 46, 60, 83 and 95, while those for the two tumors in the sh#45 group were 23 and 67.) **(B)** Efficient knockdown of LGR5 was established in Huh-7 cells using sh#86 as compared to the non-target control (NTC). This suppressed sphere formation *in vitro* significantly and inhibited tumorigenicity *in vivo*.

As Dvl is an important mediator of the Wnt signaling pathway [21] which is implicated in cancer stem cell maintenance in other cancers [22–25], we examined the role of Dvl3 in regulating the stemness features in HCC. We observed that knockdown of Dvl3 significantly suppressed the sphere formation ability of both Huh-7 and MHCC-97L HCC cell lines (*P* = .037 and .008, respectively) as compared to the non-target control (NTC) (Figure 5A and Supplementary Figure 3A). Furthermore, knockdown of Dvl3 significantly suppressed the expression of stemness genes (Supplementary Figure 3B) and enhanced the chemo-sensitivity towards the chemodrug cisplatin as compared with the NTC (*P* = .005 and .016 for Huh-7 and MHCC-97L, respectively) (Supplementary Figure 3C). For the effect of Dvl3 knockdown on *in vivo* tumorigenicity, 5 × 10^4^ and 5 × 10^5^ cells were injected into NOD-SCID mice. Dvl3 knockdown in Huh-7 cells reduced the incidence of tumor formation *in vivo* as compared to the NTC group (Figure 5A). A similar trend was observed in Dvl3-knockdown MHCC-97L cells, and there was a reduction in the incidence of tumor formation with injection of 5 × 10^4^ Dvl3-knockdown cells as well as a delay in tumor onset (Supplementary Figure 3A). It was also noted that the knockdown of Dvl3 did not significantly suppress the exponential growth rate of individual tumors (Supplementary Figures 3D and 3E) as we monitored the sizes of individual tumors throughout the course of the experiment.

As we demonstrated that Dvl3 upregulated LGR5 expression in HCC cells (Supplementary Figure 2A), we also investigated the role of LGR5 in HCC stemness. Stable knockdown of LGR5 with shLGR5 #86 suppressed the sphere formation ability in both Huh-7 and MHCC-97L cells (*P* = .034 and *P* = .001, respectively) (Figure 5B and Supplementary Figure 3F). Those cells with significantly higher LGR5 expression as obtained from FACS showed significantly enhanced sphere forming ability (Supplementary Figures 2F, 2G and 3G). Furthermore, knockdown of LGR5 suppressed the tumor formation incidence in NOD-SCID mice in both Huh-7 and MHCC-97L (Figure 5B and Supplementary Figure 3G). The knockdown clones showed delayed tumor onset for Huh-7 and more so for MHCC-97L when 5 × 10^4^ and 5 × 10^5^ cells were injected. Lastly, in addition to the finding that HIPK2 promoted NP-Dvl3 to enhance LGR5 expression in HCC cells, we observed that functionally, HIPK2, Dvl3 and LGR5 promoted HCC stemness. With stable knockdown approach, knockdown of HIPK2 significantly suppressed sphere formation (Supplementary Figure 3H).

## DISCUSSION

In this study, we found that Dvl3 expression was upregulated in human HCCs. We have shown that Dvl3 promoted cancer stemness in terms of enhanced tumorigenicity, sphere formation ability, chemoresistance, and expression of stemness genes. However, how its stability is controlled has not been previously clarified.

When Dvl3 is phosphorylated upon Wnt stimulus, it appears as a more slowly migrating band (P-Dvl3) as compared to the faster migrating band (NP-Dvl3) in Western blots [6, 7, 26–29], and hence P-Dvl3 was believed to be the active form in the past. However, this notion has recently been debated. First, Dvl phosphorylation occurs after the activation and accumulation of β-catenin [27]. Second, hyper-phosphorylation of Dvl by overexpressing CK1ε does not result in the punctate distribution of Dvl which is characteristic of Wnt signaling activation [6, 26]. Third, mutating the phosphorylation sites at the C-terminal tail of Dvl2 enhances Wnt/β-catenin signaling [6]. Therefore, P-Dvl may not be the active form and its appearance may just represent the legacy of an already activated Wnt pathway. A recent study further showed that HIPK2 facilitates PP1Cα to de-phosphorylate Dvl1 protein in HeLa cells and zebra fish embryo to promote the stabilization of the Dvl1 protein [7]. Also, the E3 ubiquitin ligase ITCH promotes the degradation of P-Dvl protein in HEK293 cells [8]. In line with these findings, our study found that the phospho-defective form of Dvl3 protein showed enhanced stability and activity on the Wnt/β-catenin signaling in HCC cells. This indicates that this phosphorylation of Dvl3 is linked to the dynamics of the amount of Dvl3 protein.

Such dynamics may represent a built-in feedback regulatory mechanism of the signaling. While Wnt ligands activate the signaling, they may also trigger Dvl phosphorylation to prime part of the Dvl protein pool to become less active. In this way, less amount of active Dvl3 will be available to convey further signals downstream to prevent prolonged activation of the pathway. This may allow time for degradation of the accumulated β-catenin to return the pathway activity to the initial basal level in order to prepare the cells for subsequent round of activation. This appears to be critical to the concise temporal and spatial Wnt-mediated biological processes.

The presence of HIPK2/PP1Cα and ITCH may offer additional layers of regulation on Dvl3 quantity and activity to help modulate the Wnt signaling pathway activity: (1) when the P-Dvl3 resulted from Wnt stimulation is de-phosphorylated by HIPK2, such Dvl3 protein can return back to the initial pool of active NP-Dvl3 to maintain sustainable Wnt signaling activity; (2) when the P-Dvl3 resulted from Wnt stimulation cannot be efficiently de-phosphorylated by the HIPK2/PP1C, this may indicate an already excessive Wnt stimulation and ITCH will remove the excessive P-Dvl3 [7, 8] in order to downsize the total Dvl3 protein pool to prevent chronic excessive signaling activity. This explains why prolonged and strong Wnt activation consumes the amount of Dvl3 protein in the cells (Supplementary Figures 4A and 4B) before newly synthesized Dvl3 protein can replenish the loss.

**Figure 6:**
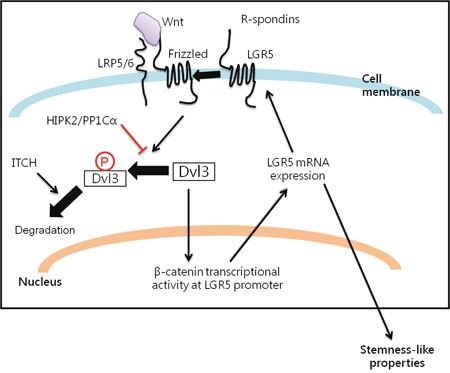
Summary of the interplay among Dvl3, HIPK2/PP1Cα/ITCH axis and LGR5 in promoting liver cancer stemness

LGR5 is a β-catenin target gene as well as a marker of crypt-locating intestinal stem cells [9, 10] and gastric stem cells [11]. Furthermore, we found that Dvl3 knockdown suppressed LGR5 while Dvl3 overexpression enhanced LGR5 expression in HCC cell lines (Supplementary Figure 2A). With these, we used the LGR5 expression as a read-out for studying the functionality of Dvl3 protein on Wnt/β-catenin signaling and stemness properties in HCC cells. In summary, our study on the Dvl3-mediated Wnt signaling pathway (Figure 6) has revealed that Dvl3 is overexpressed in human HCCs. The NP-Dvl3 is the more stable and active form of the protein and enhances HCC stemness. HIPK2/PP1Cα prevents Wnt3a-induced phosphorylation of Dvl3 and preserves the activity of Dvl3. This also spares the Dvl3 protein from degradation by ITCH if remaining in the phosphorylated state otherwise. Altogether, HIPK2/PP1Cα and ITCH maintain the balance of Wnt/β-catenin signaling activity. Disturbance to such balance (e.g. by an increase in HIPK2 expression) may lead to aberration of Wnt/β-catenin signaling activity and subsequent upregulation of β-catenin downstream target such as LGR5 to promote the Dvl3-driven hepatocarcinogenesis.

## MATERIALS AND METHODS

### Clinical samples and cell lines

All HCC specimens and their corresponding non-tumorous liver tissues were resected from Chinese patients between year 1992 to 2000 at Queen Mary Hospital, Hong Kong. Thirteen patients were male and seven were female. Their ages ranged from 38 to 71 years (mean = 56.1 years). Sixteen (80%) of the 20 patients had chronic hepatitis B viral infection with positive serum hepatitis B surface antigen (HBsAg) status. None of these patients received any other therapies including chemoembolization or chemotherapy prior to hepatic tumor resection. Following surgical resection, all specimens were either snap-frozen immediately in liquid nitrogen and stored at −80°C, or fixed in buffered 10% formalin for paraffin embedding. The use of the clinical specimens was approved by the Institutional Review Board of the University of Hong Kong/Hospital Authority. Human HCC cell lines (Hep3B, HLE, Huh-7, PLC/PRF/5), human embryonic kidney cells HEK-293, human hepatoblastoma cell line HepG2, mouse L-cells and Wnt3a-producing L-cells were obtained from American Type Culture Collection. MHCC-97L was a gift from Dr. Z.Y. Tang of Fudan University, Shanghai. BEL7402, SMMC-7721, LO2 and MIHA were obtained from Shanghai Institute of Cell Biology, Chinese Academy of Sciences. All cell lines were maintained as described [30].

### Plasmids, cell transfection and establishment of stable clones

The Dvl3-expressing construct (Dvl3/pcDNA) was a gift from Dr M. Semenov (Harvard University, MA). The N-terminally Flag-tagged and myc-tagged Dvl3 was cloned from Dvl3/pcDNA to FLAG/pcDNA3.1 and 2x-myc/pcDNA3.1 plasmids respectively. Phospho-defective mutant of Dvl3 (P2A) was created using mutational primers 5′-AGCGAAGGCAGTCGGAGCAGTGGCGCCAACCGTGCCGGCAGCGATCGGAGG-AAGGAGAAG and 5′-CTTCTCCTTCCTCCGATCGCTGCCGGCACGGTTGGCGCCA- CTGCTCCGACTGCCTTCGCT. The N-terminally GFP-tagged HIPK2 was made by cloning HIPK2 to pEGFP-C1 plasmid. The N-terminally FLAG-tagged HIPK2 constructs were gifts from Dr. L. Schmitz (University of Giessen, Germany). The N-terminally myc-tagged ITCH was created by cloning ITCH to 2x-myc/pcDNA3.1. Super8X TOP Flash and Super8X FOP Flash constructs were gifts from Dr. R. Moon (University of Washington, Seattle, WA) for TOP/FOP reporter assays. pRL-CMV construct with a CMV promoter-driven renilla luciferase reporter was transfected as reference for normalization. LGR5 promoter was cloned to pGL3-Basic plasmid. Small interfering RNA (siRNA) targeting Dvl3 was purchased from Dharmacon, Inc while those targeting HIPK2, PP1Cα and ITCH were from Integrated DNA Technologies, Inc. based on sequence available from literature.[7, 8] Transfection was done using Lipofectamine 2000 according to the manufacturer's instruction. Dvl3, LGR5 and HIPK2 knockdown cells were established by lentiviral system using the MISSION® short hairpin RNA (shRNA) constructs (Sigma-Aldrich, St. Louis, Missouri) under selection in appropriate concentration of puromycin according to the manufacturer's instruction.

### RNA extraction and quantitative reverse-transcription polymerase chain reaction (RT-qPCR)

TRIzol reagent (Invitrogen, Carlsbad, CA) and Reverse-Transcription kit (Qiagen, Venlo, Netherlands) were used to extract total RNA and generate complementary DNA respectively according to the manufacturers' protocols. RT-qPCR was carried out using Taqman probes for Dvl3 and HPRT (Applied Biosystems, Carlsbad, CA) in the 7900HT Fast Real-Time PCR system (Applied Biosystems). SYBR green RT-qPCR was used to analyze gene expressions in cell lines. The list of primers is shown in Supplementary Table 1.

### Protein preparation, co-immunoprecipitation (Co-IP) and western blot analyses

Lysate preparation and SDS–polyacrylamide gel electrophoresis for Western blotting were performed as described [31, 32]. Antibodies for Dvl3 (Cell Signaling Biotechnology, Danvers, MA and Santa Cruz Biotechnology, Dallas, Texas), Flag-tag (Sigma-Aldrich, St. Louis, MO), myc-tag (Santa-Cruz Biotechnology), GFP (Santa-Cruz Biotechnology), β-catenin (BD Bioscience, Franklin Lakes, NJ), PP1Cα (Santa Cruz Biotechnology), ITCH (Cell Signaling Biotechnology) and β-actin (Sigma-Aldrich) were used. Band densitometry on Western blots was calculated using AlphaEaseFC^TM^ software (Genetic Technologies, Miami, FL). Co-IP was performed in Huh-7 cells at 48 hours after transfection, as previously described [33].

### Sphere formation assay

Briefly, cells were suspended in 0.25% methyl cellulose in serum-free DMEM/F12 in wells pre-coated with 1% polyHEMA to form spheres in presence of the necessary supplement as described [34]. At end point, the numbers of spheres were counted.

### Fluorescence-activated cell sorting (FACS)

The LGR5 promoter-driven cyan fluorescent protein (CFP)-containing construct was transfected to BEL-7402 cells and cell sorting was performed as described [34] 48 hrs after the transfection.

### Annexin V assay

Commercial kit (BD Biosciences, Sparks, MD) was used according to the manufacturer's instruction. By using a FACSCalibur flow cytometer and CellQuest software (BD Biosciences), 10,000 events were acquired per sample under proper gating for PI and annexin V signals for analysis.

### *In vivo* tumorigenicity assay

To examine the self-renewal ability of the injected cells to generate tumors in immunodeficient mice [35], indicated numbers of cells were injected subcutaneously to the flanks of different groups of male NOD-SCID mice of 4-5 weeks of age. Latency for tumor occurrence and tumor incidence for different groups of mice were recorded.

### Dual luciferase reporter assay

TOP/FOP reporter assays were performed [36] using the Dual-Luciferase® Reporter (DLR™) Assay System (Promega, Fitchburg, Wisconsin) to determine β-catenin transcriptional activity, according to the manufacturer's protocol. Wnt3a L-cell conditioned medium and L-cell conditioned medium were collected as described [37].

### Statistical analyses

Chi-squared test or Fisher's exact test was used to analyze categorical data, while Student's *t* or Mann–Whitney test was used for continuous data. Tests were considered significant when the *P*-value was < 0.05.

## SUPPLEMENTARY FIGURES AND TABLE



## Abbreviations

HCC: hepatocellular carcinoma
Dvl3: Dishevelled-3
NP-Dvl3: non-phosphorylated Dvl3
HIPK2: homeodomain-interacting protein kinase 2
PP1Cα: protein phosphatase regulatory unit C-alpha
ITCH: itchy E3 ubiquitin ligase
LGR5: leucine-rich repeat-containing G-protein coupled receptor
DIX domain: dishevelled and axin domain
PDZ domain: post synaptic density-95, discs Large, and zonula occludens-1 domain
DEP domain: dishevelled, EGL-10, Pleckstrin domain
LC: L cell control
P2A: phospho-defective mutant of Dvl3
GFP: green fluorescent protein
siRNA: small interfering RNA
shRNA: short hairpin RNA
WT: wild-type
D45: serine 45 deletion mutant of β-catenin
GSK3: glycogen synthase kinase 3
RT-qPCR: real-time quantitative polymerase chain reaction
Co-IP: co-immunoprecipitation
SDS-PAGE: sodium dodecyl sulfate-polyacrylamide gel electrophoresis
NTC: non-target control
P-Dvl3: phosphorylated Dvl3
Wnt3a CM: Wnt3a conditioned medium
BMI1: B lymphoma Mo-MLV insertion region 1
EpCAM: epithelial cell adhesion molecule
HPRT: hypoxanthine-guanine phosphoribosyltransferase
RLU: relative luciferase intensity unit
mRNA: messenger RNA
exp: exposure
β-cat: β-catenin
