# Supplementary material for: Dishevelled-3 phosphorylation is governed by HIPK2/PP1Cα/ITCH axis and the non-phosphorylated form promotes cancer stemness via LGR5 in hepatocellular carcinoma
Source: Oncotarget. 2017 Apr 11;8(24):39430–42. doi: 10.18632/oncotarget.17049 (PMC5503623; doi:10.18632/oncotarget.17049)
Supplement: Supplementary file 1 [file oncotarget-08-39430-s001.pdf]

Dishevelled-3 phosphorylation is governed by HIPK2/PP1Cα/ITCH axis and the non-phosphorylated form promotes cancer stemness via LGR5 in hepatocellular carcinoma

SUPPLEMENTARY FIGURES AND TABLE

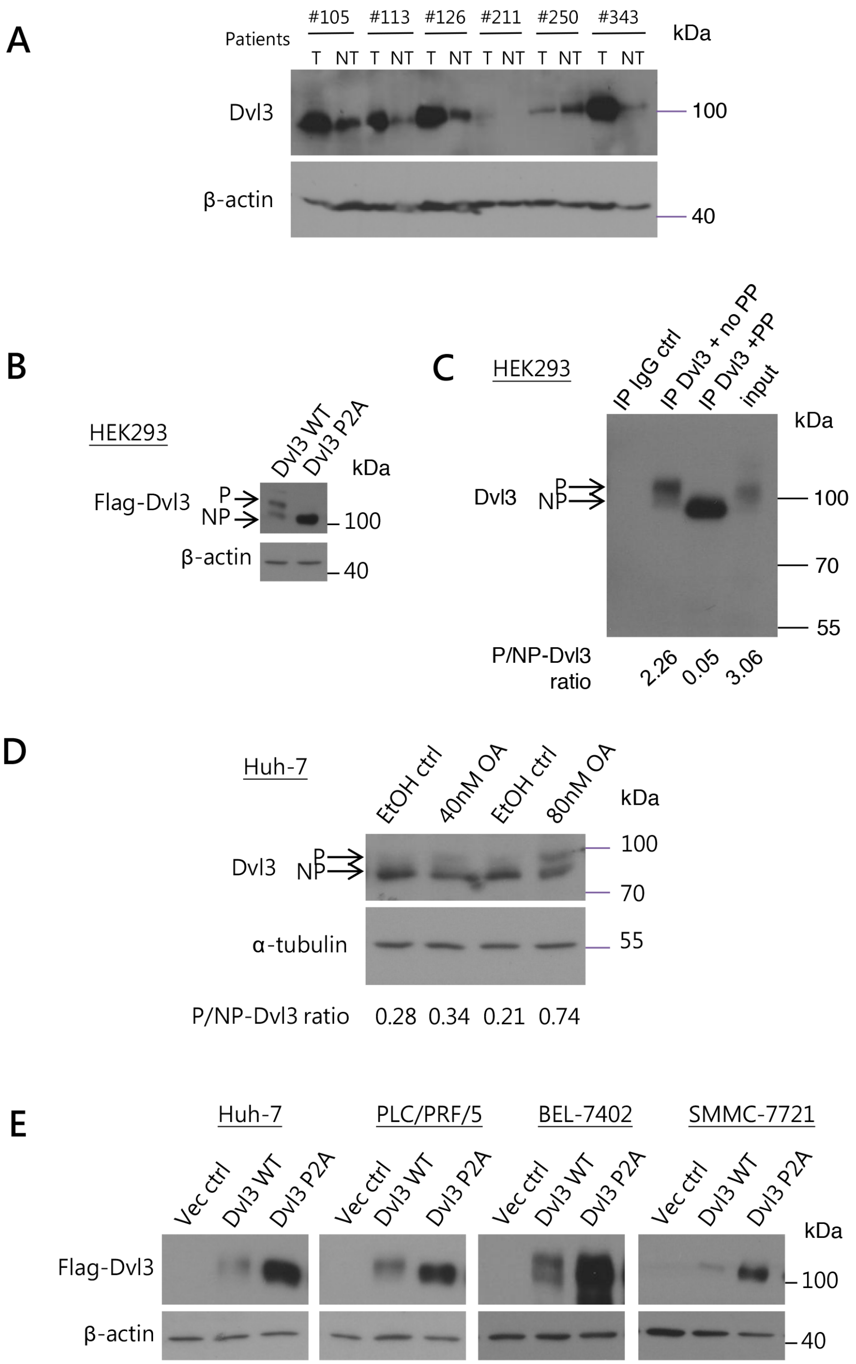

(Continued)

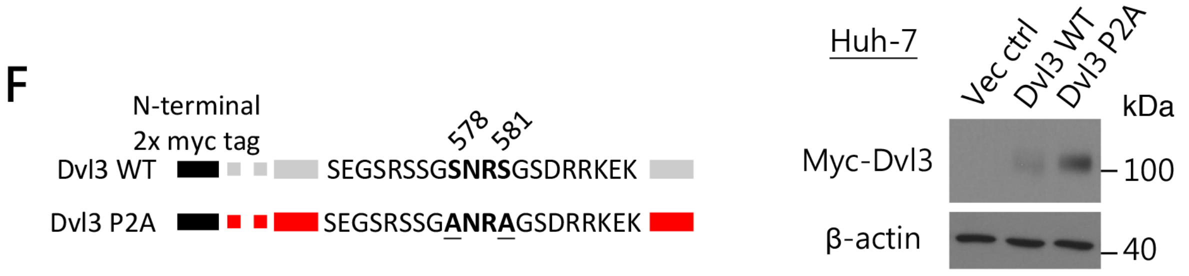

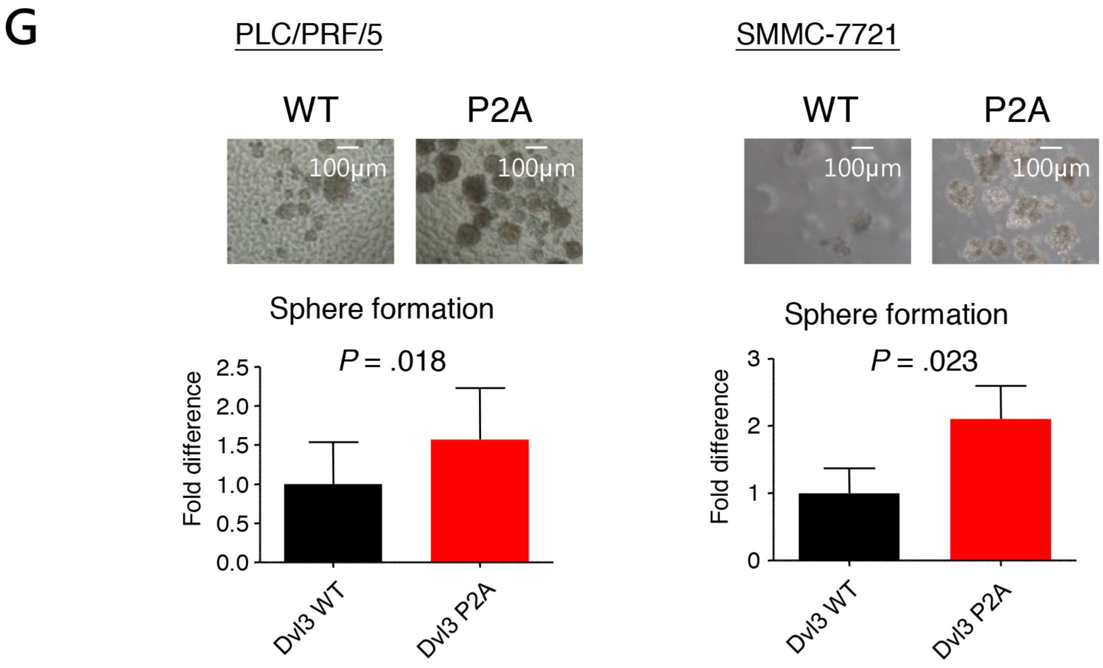

Supplementary Figure 1 (*Continued*): Non-phosphorylated Dvl3 is the more stable and active form of Dvl3 in HCC cells. (A) Western blotting for Dvl3 protein levels in HCC tumors (T) and the corresponding non-tumors (NT) were performed. Representative cases are shown. β-actin was used for normalization. **(B)** Mutation of the phosphorylation sites was demonstrated by Western blot showing phosphorylated (P) and non-phosphorylated (NP) Dvl3 in HEK293 cells. **(C)** Dvl3 protein was pulled down by anti-Dvl3 antibody in HEK293 cell lysate. This pull-down fraction was then subjected to phosphatase (PP) treatment (50U/20μl) or buffer-only (no PP) treatment respectively at 37°C for 1hr. Western blot was performed and densitometry for the upper and the lower bands was determined. Phosphatase treatment clearly removed the upper band as compared to the no treatment control, indicating that the upper and lower bands indeed corresponded to the P- and NP-Dvl3 respectively. **(D)** Treatment of okadaic acid (OA), a phosphatase inhibitor, for 4hrs, increased the ratio of densitometry for the upper to the lower bands in a dose-dependent manner in Huh-7 cells. Ethanol (EtOH) was the solvent for okadaic acid and used as the blank control here. **(E)** The Flag-tagged phospho-defective Dvl3 mutant (P2A) showed increased protein stability than the wild-type (WT) in most HCC cell lines, including Huh-7, PLC/PRF/5, BEL-7402 and SMMC-7721. Same amount of DNA constructs were transfected across vector control, WT and the P2A mutant. **(F)** The non-phosphorylated Dvl3 mutant showed increased protein stability than the wild-type (WT) in HCC cells. The Dvl3 protein was myc-tagged at N-terminal. Same amount of DNA constructs were transfected across vector control, WT and the P2A mutant into Huh-7 cells. **(G)** The NP-Dvl3 mutant promoted greater sphere forming ability than WT Dvl3 in PLC/PRF/5 and SMMC-7721 cell lines. All *in vitro* experiments were carried out in at least 3 independent trials and the values are represented as mean ± SD.

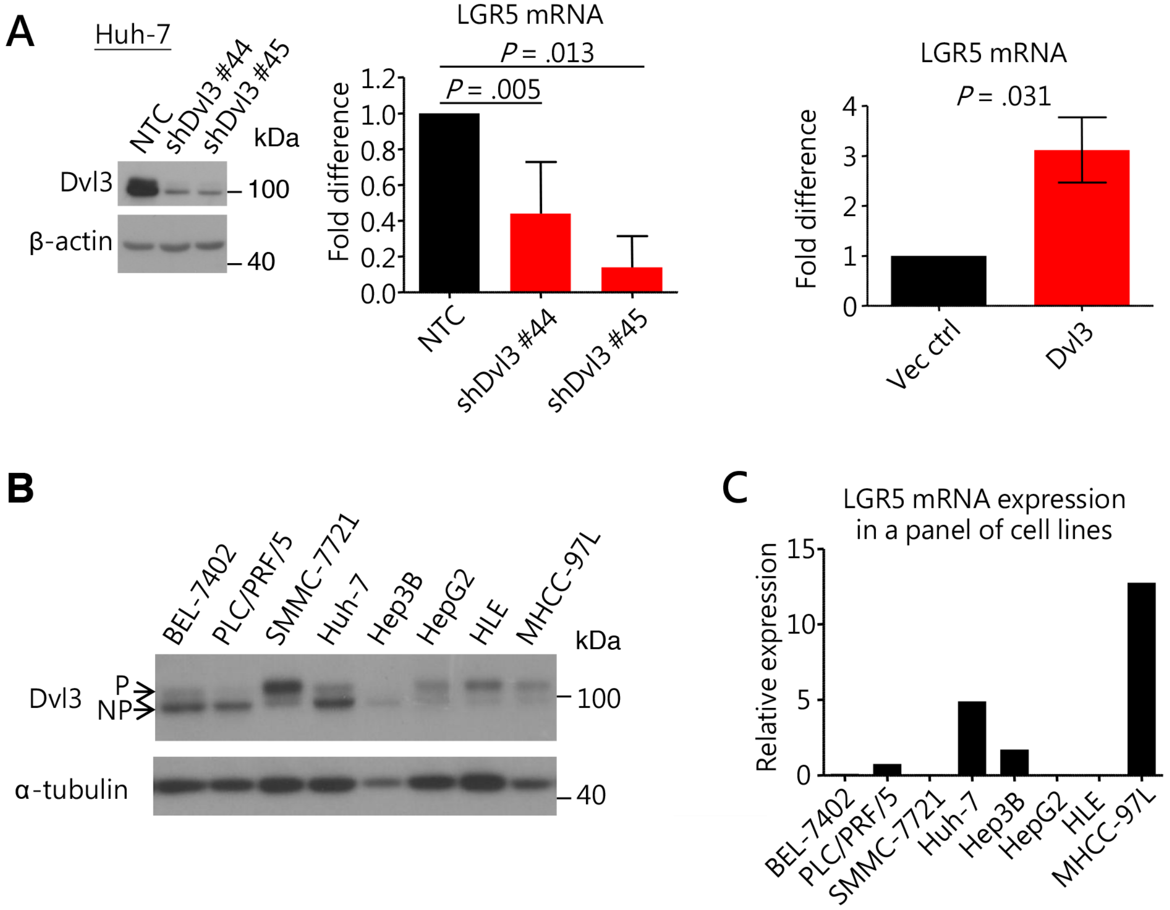

D

Summary of Dvl3 protein amount and LGR5 mRNA in cell line panel

	BEL-7402	PLC/PRF/5	SMMC-7721	Huh-7	Hep3B	HepG2	HLE	MHCC-97L
NP	0.51	0.45	0.24	0.54	0.19	0.12	0.09	0.15
P	0.22	0.10	0.66	0.24	0.02	0.19	0.26	0.29
NP / P	2.39	4.39	0.37	2.23	9.88	0.65	0.34	0.51
NP>P	Yes	Yes	No	Yes	Yes	No	No	No
LGR5 mRNA	0.10	0.75	0.07	4.90	1.71	0.07	0.06	12.76
LGR5 mRNA > median	No	Yes	No	Yes	Yes	No	No	Yes

(*Continued*)

E

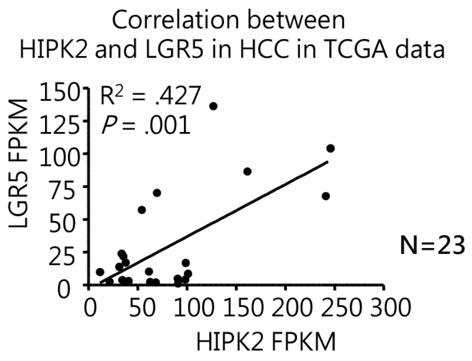

F

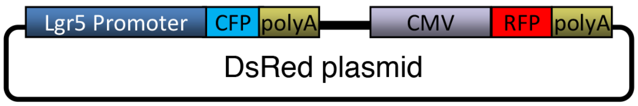

G BEL-7402

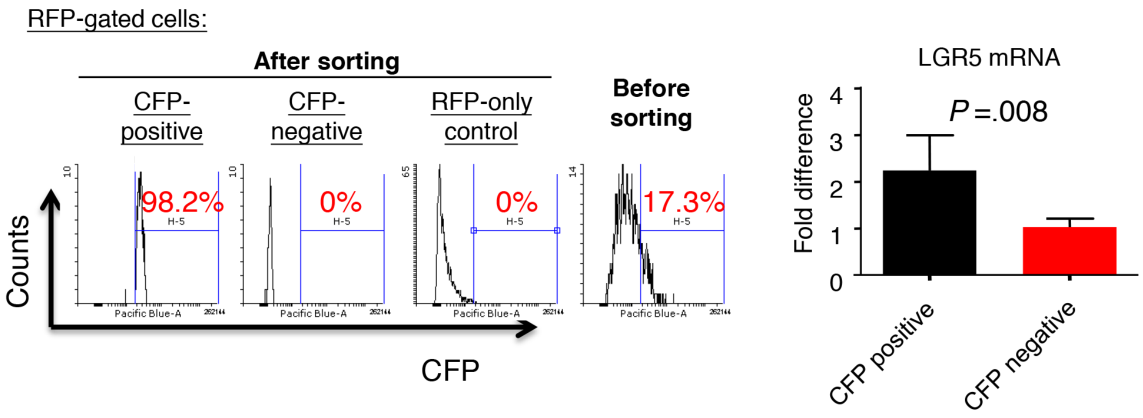

Supplementary Figure 2 (*Continued*): Non-phosphorylated Dvl3 as mediated by HIPK2/PP1Cα helps sustain LGR5 mRNA expression in HCC. (A) Knockdown of Dvl3 in Huh-7 suppressed the expression of LGR5 when compared to the NTC; while transient transfection of Dvl3-pcDNA3.0 construct significantly increased LGR5 expression as compared to the vector control. **(B)** Western blot for cell panel of phosphorylated (P) and non-phosphorylated (NP) Dvl3 protein. **(C)** Cell panel of LGR5 mRNA expression relative to 1000 β-actin as determined by RT-qPCR. **(D)** Table summarizing Dvl3 protein phosphorylation status and amounts and LGR5 mRNA expression in a panel of HCC cell lines. **(E)** Significant correlation of HIPK2 and LGR5 in HCC tumors with detectable LGR5 mRNA expression according to the data from The Cancer Genome Atlas (TCGA) (N=23). **(F)** Constructs containing LGR5 promoter driven cyan-fluorescent protein (CFP) and CMV promoter-driven red-fluorescent protein (RFP). The latter was used to label the successfully transfected cells. **(G)** By fluorescence-activated cell sorting (FACS), the BEL-7402 cells with CFP signal to indicate LGR5 expression and those without were sorted out *(left)*. Successful cell sorting was verified by significantly higher LGR5 mRNA expression in CFP-positive cells than CFP-negative cells *(right)*.

A

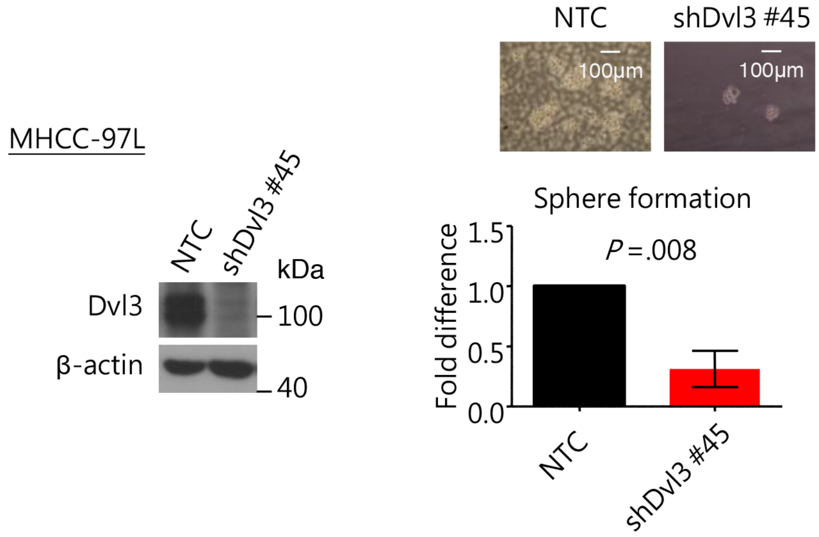

No. of cells injected	Tumor incidence		Median no. of days of tumor onset	
	NTC	shDvl3 #45	NTC	shDvl3 #45
5 x 10^4	7 / 14	2 / 14	31	66
5 x 10^5	2 / 2	2 / 2	10	20

B

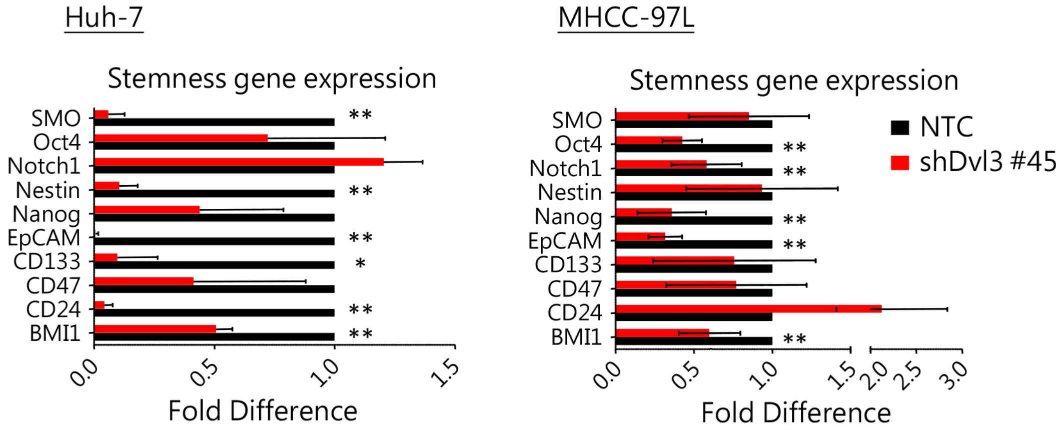

(*Continued*)

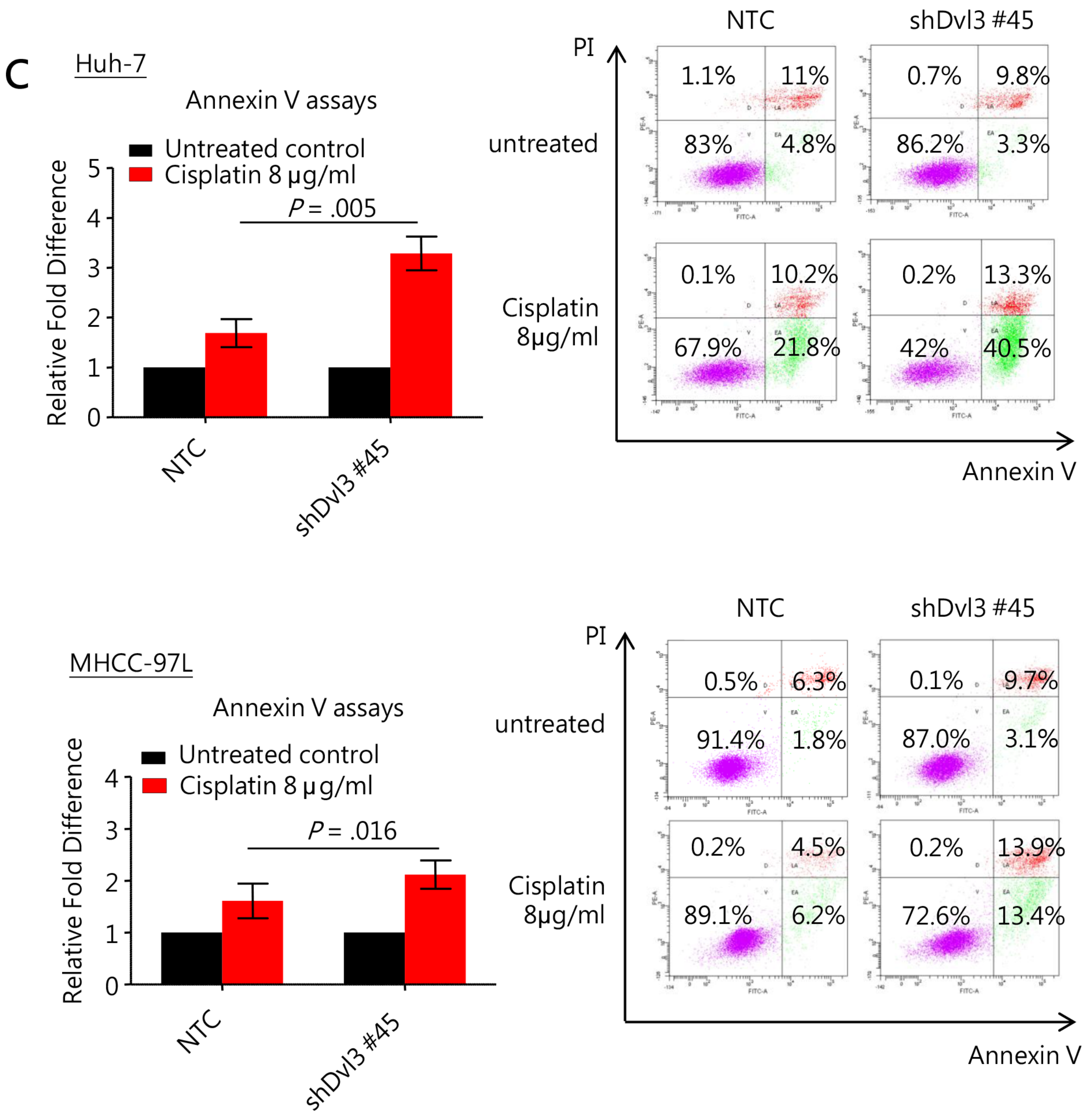
C
Huh-7
Annexin V assays
Untreated control
Cisplatin 8 μg/ml
P = .005
Relative Fold Difference
0
1
2
3
4
5
NTC
shDvl3 #45
NTC
shDvl3 #45
PI
untreated
1.1%
11%
83%
4.8%
0.7%
9.8%
86.2%
3.3%
Cisplatin 8μg/ml
0.1%
10.2%
67.9%
21.8%
0.2%
13.3%
42%
40.5%
Annexin V
MHCC-97L
Annexin V assays
Untreated control
Cisplatin 8 μg/ml
P = .016
Relative Fold Difference
0
1
2
3
4
NTC
shDvl3 #45
NTC
shDvl3 #45
PI
untreated
0.5%
6.3%
91.4%
1.8%
0.1%
9.7%
87.0%
3.1%
Cisplatin 8μg/ml
0.2%
4.5%
89.1%
6.2%
0.2%
13.9%
72.6%
13.4%
Annexin V

(*Continued*)

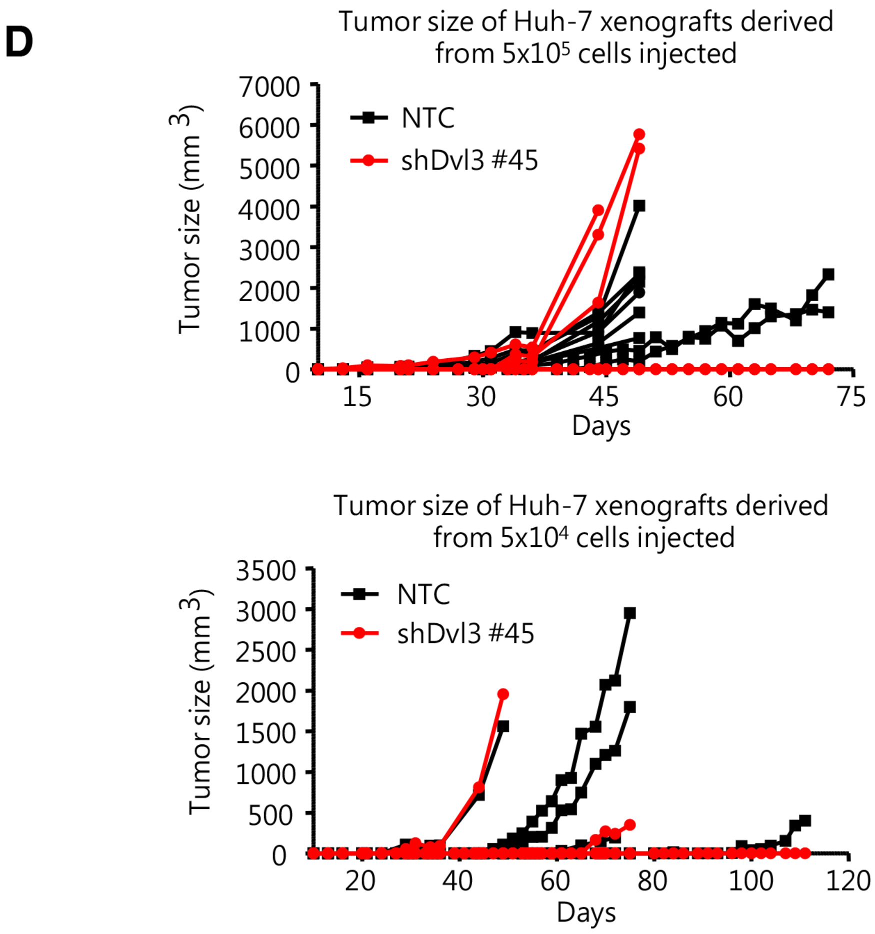
D
Tumor size of Huh-7 xenografts derived from 5x10⁵ cells injected
Tumor size (mm 3)
NTC
shDvl3 #45
Days
Tumor size of Huh-7 xenografts derived from 5x10⁴ cells injected
Tumor size (mm 3)
NTC
shDvl3 #45
Days

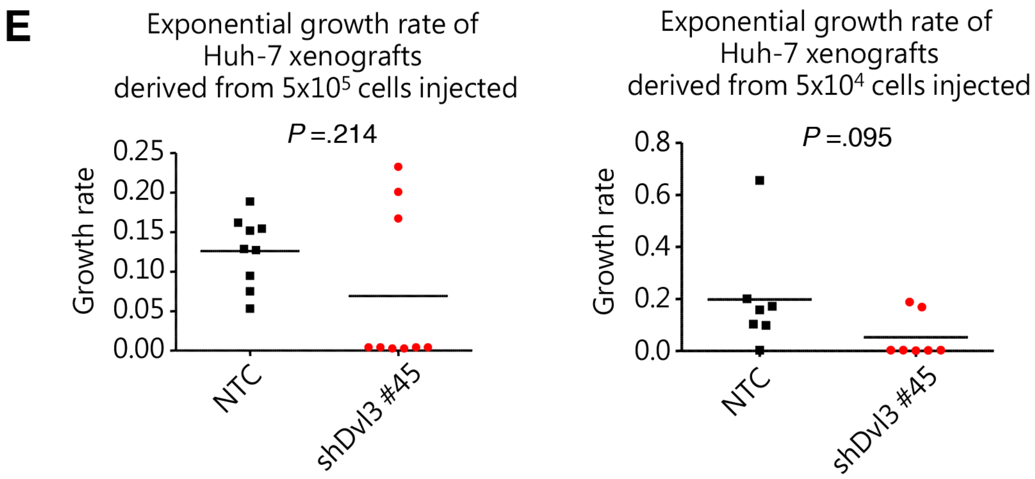
E
Exponential growth rate of Huh-7 xenografts derived from 5x10⁵ cells injected
P =.214
Growth rate
NTC
shDvl3 #45
Exponential growth rate of Huh-7 xenografts derived from 5x10⁴ cells injected
P =.095
Growth rate
NTC
shDvl3 #45

(*Continued*)

F

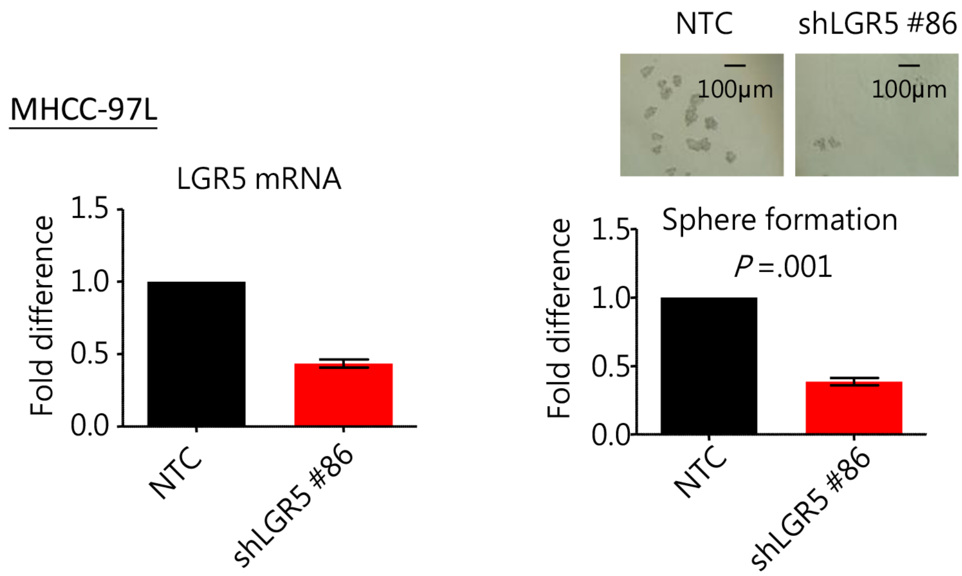

No. of cells injected	Tumor incidence		Median no. of days of tumor onset	
	NTC	shLGR5 #86	NTC	shLGR5 #86
5 x 10^3	9 / 14	4 / 14	76^	69^
5 x 10^4	12 / 14	11 / 14	24	36
5 x 10^5	9 / 9	7 / 9	10	24

G

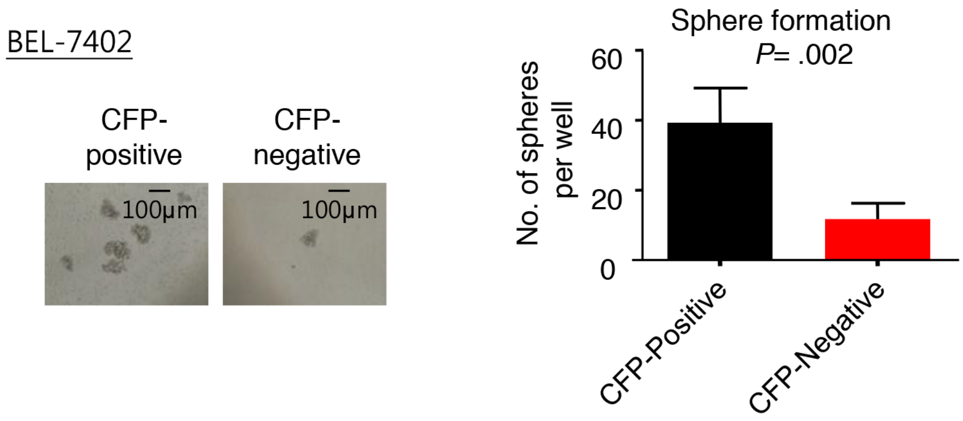

(*Continued*)

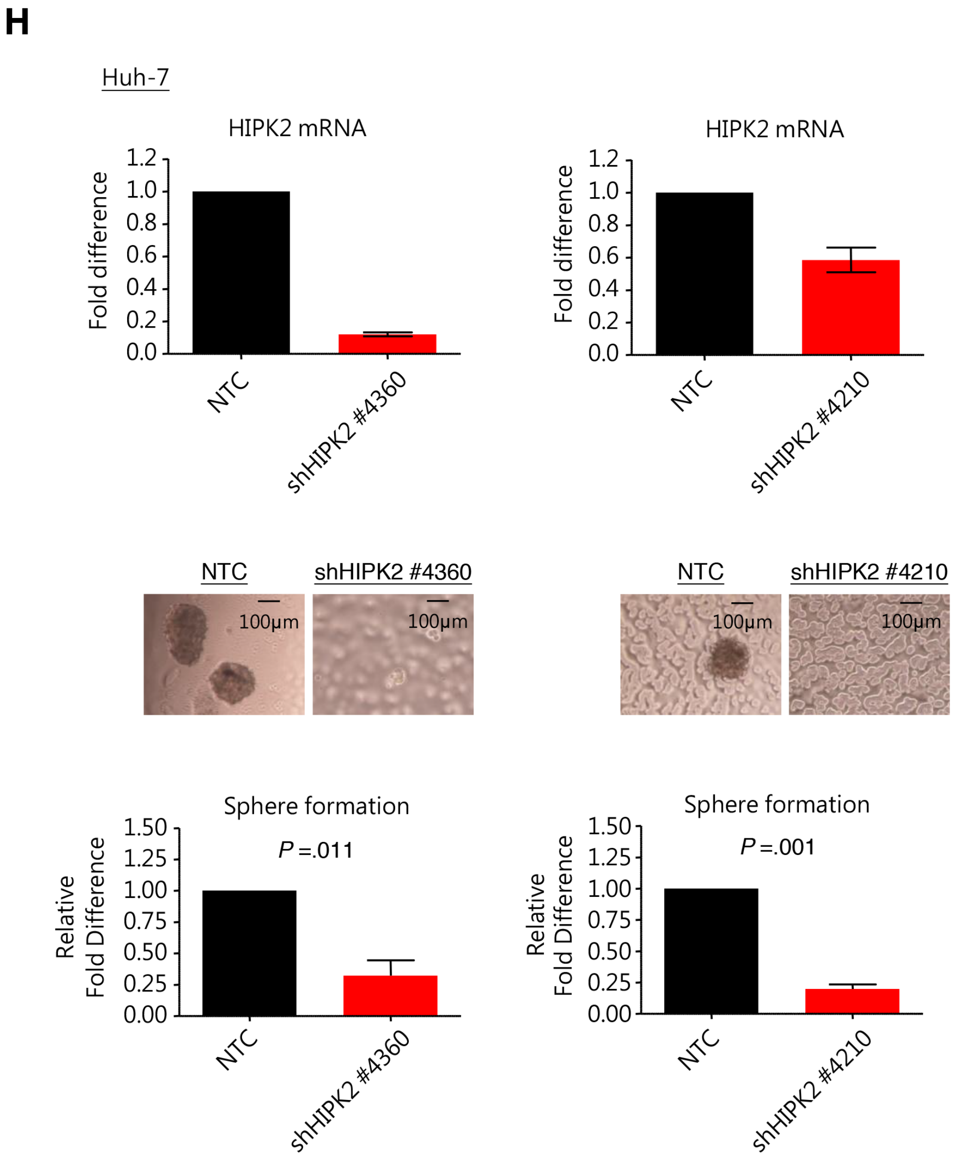

Supplementary Figure 3 (*Continued*): Knockdown of Dvl3 and LGR5 suppresses stemness properties in HCC cells. **(A)** Efficient knockdown of Dvl3 was established in MHCC-97L human HCC cell lines using sh#45 as compared to the non-target control (NTC). Knockdown of Dvl3 with sh#45 significantly suppressed sphere formation ability *in vitro*. It also inhibited tumorigenicity at $5x10^4$ cells injected and delay tumor onset in the MHCC-97L cells. **(B)** Knockdown of Dvl3 with sh#45 significantly suppressed the expression of many stemness genes in Huh-7 and MHCC-97L cells. **(C)** Dvl3 knockdown suppressed chemoresistance of HCC cells. Knockdown of Dvl3 by sh#45 significantly enhanced apoptosis upon cisplatin treatment (8μg/ml, 48h) as compared to NTC in both Huh-7 and MHCC-97L cells. Flow cytometry charts of annexin V assays are shown in the right panel. **(D)** Sizes for all tumors were monitored and recorded throughout. Each single line represents the growth curve of the respective individual tumor for the non-target control (NTC) and the Dvl3-knockdown down (sh#45) group. The upper and the lower panel showed the growth curves for the groups injected with $5x10^5$ and $5x10^4$ Huh-7 cells respectively. **(E)** Exponential growth rates of tumor sizes were calculated. Mann Whitney tests were performed. There was no significant difference in the growth rates between the non-target control (NTC) and the Dvl3-knockdown down (sh#45) groups for both the $5x10^5$ and $5x10^4$ Huh-7 cells-derived tumor xenografts. **(F)** Effective LGR5 knockdown with sh#86 significantly suppressed sphere formation in MHCC-97L. It also reduced tumor incidence and delayed onset of tumor, particularly at $5x10^4$ and $5x10^5$ cells injected, in NOD-SCID mice. The median number of days of tumor onset was calculated based on those successfully formed tumors. (^ The numbers of days after the injection for the onset of the nine tumors in the NTC group were 54, 58, 64, 70, 76, 81, 89, 98 and 116, while those for the four tumors in the sh#86 group were 58, 62, 76 and 93.) **(G)** Sphere formation was significantly higher in LGR5-high cells than LGR5-low cells. LGR5 promoter driven cyan-fluorescent protein (CFP) was used to indicate LGR5 expression and by fluorescence-activated cell sorting (FACS), the BEL-7402 cells with CFP signals and those without were sorted out. **(H)** Efficient knockdown of HIPK2 was established in Huh-7 cell line using sh#4360 and sh#4210, respectively, as compared with NTC. Stable knockdown of HIPK2 suppressed sphere formation. **(A)-(H)** Data from all *in vitro* experiments were obtained from at least 3 independent experiments, and the values are represented as mean ± SD. *$P<.050$, **$P<.010$.

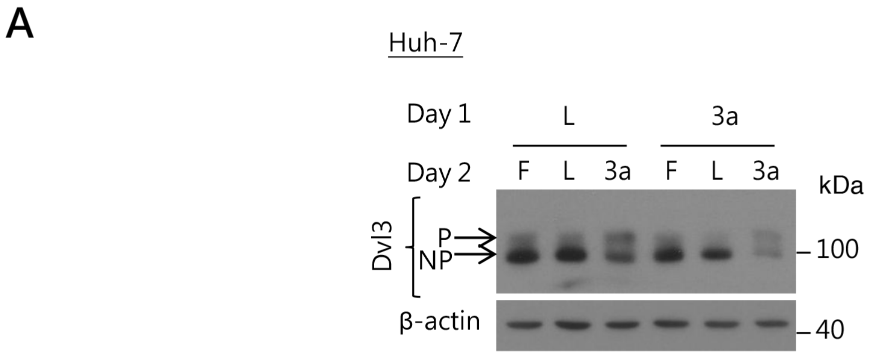

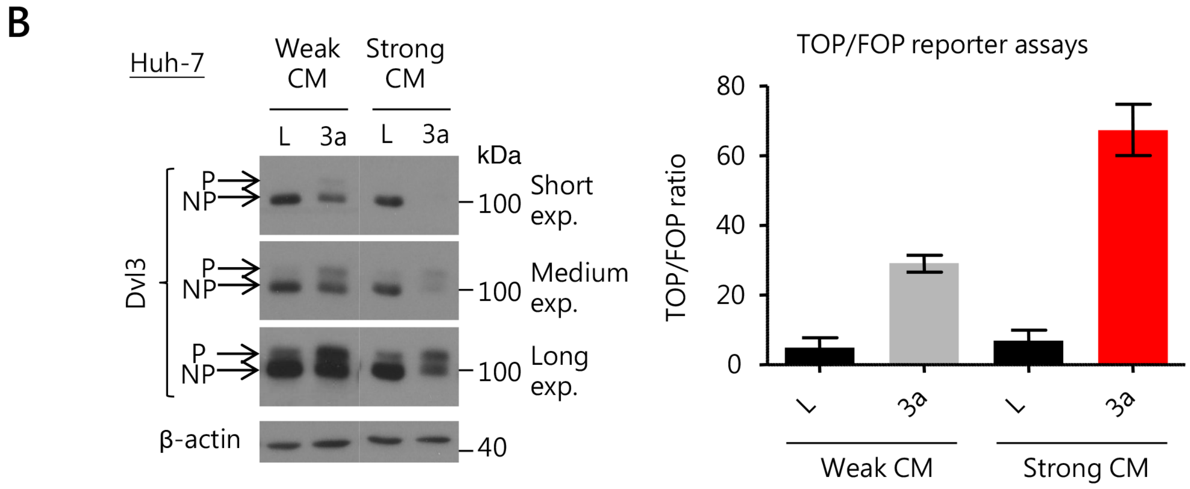

Supplementary Figure 4: Wnt3a addition consumes Dvl3 protein in HCC cells. (A) Longer exposure of Wnt3a conditioned medium (CM) stimulation resulted in faster consumption of Dvl3 protein in Huh-7. L-cell control medium (L) and blank medium (F) served as control here. **(B)** Stronger Wnt3a conditioned medium (CM) stimulation resulted in faster consumption of Dvl3 protein in Huh-7 *(left)*. L-cell control medium (L) served as control here. The strength of the Wnt3a CM has been validated by the accumulative luciferase activity in TOP/FOP dual luciferase reporter assays *(right)*.

Supplementary Table 1: List of primers used in RT-qPCR in the study

Genes	Forward primers	Reverse primers
BMI1	5’ TGGACTGACAAATGCTGGAG	5’ GTACTGGGGCTAGGCAAACA
β-actin	5’ GTGGGGCGCCCCAGGCACCA	5’ CTCCTTAATGTCACGCACGATTTC
β-catenin	5’ ATGGCCATGGAACCAGACAG	5’ GATTGCACGTGTGGCAAGTT
CD24	5’ TGAAGAACATGTGAGAGGTTTGAC	5’ GAAAACTGAATCTCCATTCCACAA
CD47	5’ CAATCACGTAAGGGTCTCATAGG	5’ GATGGACTCCGATTTGGAGA
CD133	5’ TGGATGCAGAACTTGACAACGT	5’ ATACCTGCTACGACAGTCGTGGT
Dvl3	5’ TCTACCACTTGGATGGGCAG	5’ GCTCCGATGGGTTATCAGCA
EpCAM	5’ CCATGTGCTGGTGTGTGAAC	5’ ACGCGTTGTGATCTCCTTCT
HIPK2	5’ CGTGCTTGGTCTTCGAAGATG	5’ GCGTGGATAAGACCTAGGCT
HPRT	5’ CTTTGCTGACCTGCTGGATT	5’ CTGCATTGTTTTGCCAGTGT
ITCH	5’ CGTCAACCACAACACACGAA	5’ GGTCCATTGTCTAGGGCAGA
LGR5	5’ CCCGAATCCCCTGCCCAGTCT	5’ TCATCCAGCCACAGGTGCCTA
Nanog	5’ GGATCCAGCTTGTCCCCAAA	5’ CAGGTCTGGTTGCTCCACAT
Nestin	5’ GGCGCACCTCAAGATGTCC	5’ CTTGGGGTCCTGAAAGCTG
Notch1	5’ TGAATGGCGGGAAGTGTGAA	5’ CACTTGTACTCCGTCAGCGT
Oct4	5’ CTTGCTGCAGAAGTGGGTGGAGGAA	5’ CTGCAGTGTGGGTTTCGGGCA
PP1Cα	5’ GACCGTGGCGTCTCTTTTAC	5’ TGGAAAGAGCACATGAGGGT
SMO	5’ TGGTCACTCCCCTTTGTCCTCAC	5’ GCACGGTATCGGTAGTTCTTGTAGC
